# Eye Dominance and Testing Order Effects in the Circularly-Oriented Macular Pigment Optical Density Measurements

**DOI:** 10.1007/s44402-026-00092-6

**Published:** 2026-04-29

**Authors:** Mukhit Kulmaganbetov, Taranjit Singh, Dmitry Pushin, Pinki Chahal, David Cory, Davis V. Garrad, Connor Kapahi, Melanie Mungalsingh, Iman Salehi, Andrew Silva, Benjamin Thompson, Zhangting Wang, Dusan Sarenac

**Affiliations:** 1 Quantum Optics Lab, Centre for Eye and Vision Research (CEVR), 17W Hong Kong Science Park, Hong Kong, PR China; 2 Department of Glaucoma, Kazakh Eye Research Institute, Almaty, Kazakhstan; 3 Entoptica Limited, Hong Kong, PR China; 4 Institute for Quantum Computing, University of Waterloo, Waterloo, Ontario Canada; 5 Department of Physics, University of Waterloo, Waterloo, Ontario Canada; 6 Incoherent Vision Inc., Waterloo, Ontario Canada; 7 School of Optometry and Vision Science, University of Waterloo, Waterloo, Ontario Canada; 8 Department of Physics, University at Buffalo, State University of New York, Buffalo, New York USA; 9 Department of Chemistry, University of Waterloo, Waterloo, Ontario Canada; 10 Department of Psychology, Idaho State University, Pocatello, Idaho USA

**Keywords:** Entoptic phenomenon, Eye dominance, Learning, Psychophysics

## Abstract

**Purpose:**

Psychophysical discrimination of structured light (SL) stimuli may be useful in screening for various macular disorders. The circularly-oriented macular pigment optical density (coMPOD), calculated from the discrimination performance of SL-induced entoptic phenomena, may reveal a novel functional biomarker of macular health. This study investigated the potential influence of eye dominance and testing order effects on SL-based stimulus perception, factors that potentially influence the sensitivity of screening tests based on SL technology.

**Methods:**

A psychophysical task was performed where various SL-based entoptic images with multiple azimuthal fringes rotating with a specific temporal frequency were projected onto the participants’ retinas (*n* = 28). By occluding the central areas of entoptic images, the retinal eccentricity *R*_*T*_ of the perceivable area of the stimuli was measured. The scale parameter of the coMPOD profile (*α*-value) was calculated for each participant using a spatiotemporal sensitivity model that takes into account the perceptual threshold measurements of SL stimuli with varying spatial densities and temporal frequencies.

**Results:**

The mean ± SD *α*-values for the dominant and non-dominant eyes were 0.11° ± 0.06° and 0.11° ± 0.05°, respectively. Similarly, the values for the first and second eyes were 0.11° ± 0.05° and 0.10° ± 0.05°, respectively. The Pearson correlation coefficient between eye dominance and testing order effects was *r* = 0.80 (*p* < 0.01). The Bland–Altman plots for both factors indicated zero bias.

**Conclusions:**

The results indicated repeatable measurements for both eyes, implying minimal impact from eye dominance and testing order on SL-based stimulus perception. The results provide a foundation for future studies exploring the clinical utility of SL tools in eye health.

Key Points

- The order of testing and which eye is dominant do not significantly affect the measurements obtained with this novel light-based macular assessment.
- The consistency of these results supports the use of this method to compare the health of a patient’s two eyes in clinics.
- This study establishes a reliable foundation for using this technology to detect early signs of macular degeneration and other eye diseases.

## Introduction

The perception of polarised light has attracted significant interest in vision science due to its potential applications in detecting and diagnosing macular diseases [1, 2]. Standard methods, such as Haidinger’s brush (HB), have been limited in effectiveness due to weak signals, low contrast, restricted stimulus flexibility and a narrow visual extent [2–6]. Using structured light (SL) techniques, light can be engineered to have distinct spatial polarisation states that elicit rich entoptic patterns and mitigate these limitations [7–9]. Notably, SL-induced entoptic phenomena with the number of azimuthal fringes (*N*_*f*_) ≥ 11 can be observed beyond the foveal region, extending up to ≈5°retinal eccentricity (*R*_*T*_) [7] compared to HB with *R*_*T*_ ≈ 2° [1, 2, 4, 6]. SL-based stimulus perception has the potential to be used as a diagnostic tool [3, 10, 11] for detecting early functional signs of macular degenerative diseases such as age-related macular degeneration (AMD) [12–14], type 2 macular telangiectasia (MacTel) [15–17] and pathologic myopia [18–20].

The ability to perceive polarisation-induced entoptic phenomena is due to the presence of dichroic macular pigments (MPs) [4, 21], which are accumulated in the inner plexiform, outer nuclear and Henle fibre layers (HFLs) of the retina [22–24]. By modulating the spatial density (*N*_*f*_) and temporal frequency (*ω*) of the azimuthal fringes and occluding areas (*R*_*T*_) in SL-based entoptic images, it is possible to characterise the circularly-oriented macular pigment optical density (coMPOD) in the radially-oriented fibres of photoreceptors in HFL [25].

SL-based tests are monocular, and clinical applications may involve comparisons between the two eyes in the same observer. Therefore, it is important to assess the impact of factors such as eye dominance and testing order on the test results. Eye dominance can involve oculomotor control [26, 27] and cortical processing [28–31]. However, an individual’s dominant eye may not always align with their optimal ability to perceive polarisation [32, 33].

Studying the impact of eye testing order aids in identifying sources of variability and determining the technique’s reliability and consistency [34–36]. The assessment of SL-based stimulus perception in a monocular context [3, 7–11, 25, 37] raises the possibility that the observer may exhibit task learning from one test to the next. Thus, it is important to ascertain potential learning effects.

## Methods

### Study Participants

A total of 35 participants (mean age 25 ± 5.5 years, range 18–38 years, 17 female/18 male) were recruited from the Centre for Eye and Vision Research. Each participant underwent a comprehensive eye and vision examination prior to inclusion, which included assessments of medical and family history, uncorrected and best-corrected visual acuity, binocular vision tests, ocular motility, contrast sensitivity, slit-lamp biomicroscopy, indirect ophthalmoscopy, colour fundus photography (Nidek AFC-330, NIDEK CO., LTD., nidek-intl.com), optical coherence tomography (Topcon DRI OCT Triton, Topcon Corporation, topconhealthcare.jp), ocular biometry (Zeiss IOLMaster 700, Carl Zeiss Meditec AG, zeiss.com) and MP optical densitometry (MPS II, IDE Vision Ltd, ide-vision.com).

Binocular vision was assessed using cover tests (cover-uncover and alternate cover) to detect strabismus, near point of convergence measurement and stereopsis assessed using the Wirt Circles (Stereo Optical Co., Inc., stereooptical.com). These tests were used to ensure normal binocular function as part of the inclusion criteria (minimum stereopsis of 40 s of arc) and were not used as covariates in the main analysis. Contrast sensitivity was assessed using the Thomson Chart (Thomson Software Solutions, thomson-software-solutions.com), which measures contrast sensitivity at spatial frequencies of 1.5, 3, 6, 12 and 18 cycles per degree. The values reported in Table 1 represent the mean log contrast sensitivity across these tested spatial frequencies. This average value was used as a generalised screening metric to ensure participants had normal overall contrast vision, rather than for the detailed analysis of specific spatial-frequency-dependent deficits.

**Table 1:** Summary of interocular differences.

Indicator	Right eye	Left eye	Paired *t*-test	
	Mean ± SD		*t*	*p* value
Uncorrected VA, logMAR	0.24 ± 0.33	0.19 ± 0.30	1.69	0.10
Best-corrected VA, logMAR	0.00 ± 0.00	0.00 ± 0.00	0	>0.99
Contrast sensitivity, log_10_	1.68 ± 0.15	1.71 ± 0.14	−1.3	0.20
HFL volume, mm^3^	2.44 ± 0.21	2.45 ± 0.19	−0.41	0.69
MPOD	0.42 ± 0.16	0.41 ± 0.13	0.49	0.63
AEL, mm	24.41 ± 1.27	24.34 ± 1.22	1.43	0.17
CCT, mm	0.54 ± 0.04	0.53 ± 0.04	1.66	0.11
ACD, mm	3.39 ± 0.36	3.4 ± 0.35	0.04	0.97
CLT, mm	3.86 ± 0.46	3.84 ± 0.46	1.57	0.13

*ACD* anterior chamber depth, *AEL* axial eye length, *CCT* central corneal thickness, *CLT* crystalline lens thickness, *HFL* Henle fibre layer, *MPOD* macular pigment optical density, *SD* standard deviation, *VA* visual acuity.

Sighting eye dominance (preference of one eye over the other when fixating on a target) was determined with the Miles (hole-in-the-hand) test. While sensory dominance (e.g., via rivalry testing) can differ from motor dominance, sighting dominance was utilised as the primary metric for ocular preference, consistent with prior literature on monocular perceptual tasks [38–40]. Sensory dominance was not assessed in this study.

Only participants with good vision (best-corrected visual acuity = 0.00 logMAR or better in each eye) and healthy eyes were included (*n* = 28), excluding those with a history of eye disease, strabismus, ocular injury, arterial hypertension or neurological disorders (*n* = 7). An a priori power analysis was conducted to determine the sample size required to detect a clinically relevant difference in the coMPOD *α*-value between eyes. Based on the variability observed in previous studies [7, 8, 25], a difference of 0.05° in the *α*-value was determined to be the minimum meaningful difference for this functional assessment. To detect this effect size with 80% power at an alpha level of 0.05 [41], a sample size of 24 participants was required. The final sample of 28 participants provided adequate power to detect clinically meaningful interocular differences.

This cross-sectional study (ClinicalTrials.gov ID: NCT05913063, June 26, 2023) adhered to the principles of the Declaration of Helsinki and received ethical approval from The Hong Kong Polytechnic University (HSEARS20210910002). Detailed study objectives and procedures were communicated to all participants, who provided written informed consent prior to data collection.

### Psychophysical Task

**Fig. 1:**
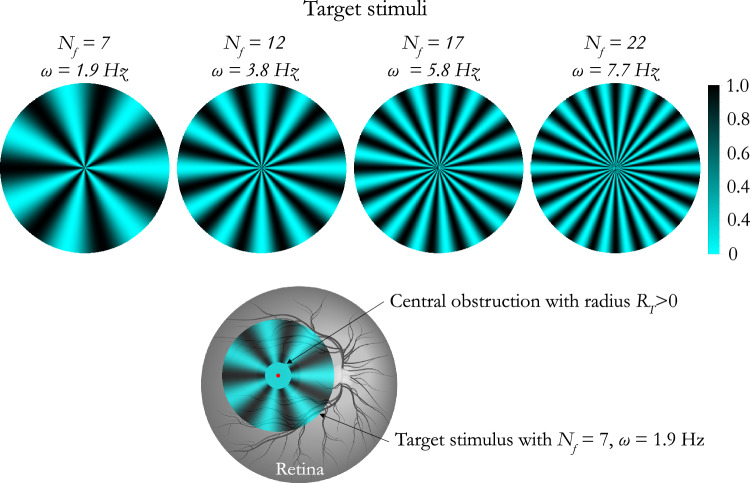
Target stimuli (top) with the number of azimuthal fringes (*N*_*f*_) = 7, 12, 17 and 22 with temporal frequency (*ω*) = 1.9, 3.8, 5.8 and 7.7 Hz, respectively, as viewed through an ideal polariser. Enface projection of the entoptic pattern with a central obstruction with radius *R*_*T*_ and *N*_*f*_ = 7 azimuthal fringes onto the retina (bottom).

The top row of Fig. 1 depicts target stimuli viewed through an ideal radial polariser. During the study, various SL-based entoptic images with visible fringe numbers *N*_*f*_ rotating with a specific temporal frequency *ω* for each *N*_*f*_ (*N*_*f*_ = 7 with *ω* = 1.9 Hz; *N*_*f*_ = 12 with *ω* = 3.8 Hz; *N*_*f*_ = 17 with *ω* = 5.8 Hz; *N*_*f*_ = 22 with *ω* = 7.7 Hz), were projected onto the retina of each participant for 0.5 s per trial. The contrast of the pattern was perceived to be highest near the centre and diminishes towards the outer edges due to the decreasing concentration of circularly-oriented MPs with retinal eccentricity [25]. The entoptic profile was rotated either clockwise or counterclockwise using a motorised polariser. Participants identified the direction of rotation of the entoptic pattern within a two-alternative forced-choice (2AFC) task. A spatial light modulator (SLM) created a circular obstruction at the centre of the entoptic image (Fig. 1, bottom). Participants were instructed to fixate on a guide light generated by aligning the centre of the stimulus, illuminated by a red laser with a maximum retinal eccentricity of 10 pixels (≈0.45°). The task required participants to perceive and discriminate the rotating entoptic fringes located in the peripheral macula (up to ≈7.5° eccentricity) outside this central foveal zone.

Similar to our previous studies [7, 25], SL techniques were used to occlude central areas of different sizes in the generated entoptic images to determine their retinal eccentricity *R*_*T*_. This threshold mask initially started from a radius (in visual degrees) of 10 pixels (≈0.45°), and if the participant could detect the correct direction of rotation of the stimuli, the threshold mask size increased with a step size of 30 pixels (≈1.35°). A 2-up/1-down staircase method aimed to estimate the size of each entoptic image that produced 71% discrimination accuracy [37]. Participants with more than one reversal point (floor performance) at the minimum obstruction radius (≈0.45°) were excluded from the analysis [7]. This type of staircase method inherently controls for random guessing. In a 2AFC task, chance performance is 50%. Consistent guessing would result in failure to reduce the obstruction radius, keeping the participant at the initial obstruction size. To proceed to changing radii and estimate a threshold, participants needed to demonstrate sustained correct discrimination. No catch trials (non-rotating stimuli) were included, as the staircase design effectively distinguishes true perception from random responses [25].

The experiment was conducted monocularly. Participants removed their standard refractive correction (spectacles/contact lenses) prior to testing to ensure the optical path of the SL was unaltered, as standard ophthalmic lenses can modify polarisation states [42, 43]. Both eyes were tested on the same day with a short break (1–2 min) between eyes. The first eye to be examined was chosen randomly, with the contralateral eye covered. The second eye was tested under the same conditions.

### Circularly-Oriented Macular Pigment Optical Density

The efficiency of the radial polariser in the human eye can be modelled by:

1 $$P\left(r\right)=1-{10}^{-M\left(r\right)}$$

where $r$ is retinal eccentricity and $M(r)$ is the profile of the coMPOD. The spatiotemporal sensitivity model introduced by Pushin et al. [25] showed that in the 1.5° to 5.5° retinal eccentricity region, the coMPOD profile *M*(*r*) is inversely proportional to *r*:

2 $$M(r)=\frac{a}{2\pi r}$$

The *α*-value is determined based on the perceptual threshold measurements of SL stimuli with different spatiotemporal frequencies and provides a unique characterisation tool for macular health. It characterises the radial scale of the retinal polarisation efficiency, determining how far from the fovea the circularly-oriented MP produces measurable polarisation contrast. Physiologically, the *α*-value sets the overall magnitude of the macular dichroic absorption across the retina. Increasing the *α*-value increases optical density at all eccentricities and therefore enlarges the retinal region that functions as an effective radial polarisation. Using Eqs. 1 and 2, the polarisation efficiency at *r* = 10*α* is approximately 3.6%. Thus, individuals with larger *α*-values retain measurable polarisation contrast farther from the fovea, whereas smaller *α*-values confine detectable polarisation signals to the central macula. This parameter, influenced by the number of Henle’s fibres and the efficiency of the radial polarisation effect of the contained MP, is essential for quantifying the visual extent of various spatiotemporal SL stimuli and individualising the *α*-value, as detailed in the model introduced in our previous study [25]. Nevertheless, it is important to note that this metric may be influenced by individual variations in macular pigment optical density (MPOD) spatial profiles, such as the presence of a foveal dip or non-exponential distribution patterns.

### Statistical Analysis

Interocular differences in functional (uncorrected and best-corrected visual acuity, contrast sensitivity) and anatomical (HFL volume, anterior chamber depth, axial eye length, central corneal and crystalline length thicknesses) indicators were compared using the paired *t*-test. Prior to applying parametric tests, the normality of data distributions was assessed using the Shapiro–Wilk test and homogeneity of variances was assessed using Levene’s test. All variables met the assumptions of normality (*p* > 0.05) and homoscedasticity, justifying the use of parametric statistical tests. The *α*-value for each eye of each participant was calculated using the threshold radius of the central obstruction for SL-induced entoptic patterns with (*N*_*f*_ = 7, *ω* = 1.9 Hz), (*N*_*f*_ = 12, *ω* = 3.8 Hz), (*N*_*f*_ = 17, *ω* = 5.8 Hz) and (*N*_*f*_ = 22, *ω* = 7.7 Hz), corresponding to an angular velocity *2πω/N*_*f*_ and was used to compute the Pearson correlation coefficient between dominant (DE) and non-dominant (NDE) eyes, as well as between the first (FE) and second (SE) eyes. The Bland–Altman method was used for visualisation of the agreement and bias between measurements from the DE vs NDE and FE vs SE by plotting the difference between the measurements against the average of the measurements.

## Results

There were no statistically significant differences observed in various ocular health indicators among the study participants (Table 1). No significant differences in the uncorrected and best-corrected visual acuity were noted. This homogeneity in the baseline measures ensures that potential confounding factors are minimised.

Of the 28 participants who completed the task, 22 were right-eye dominant and 6 were left-eye dominant. The average threshold radius (*R*_*T*_) for the SL-based entoptic phenomena with *N*_*f*_ = 7, 12, 17 and 22 are presented in Table 2 for the DE and NDE, while Table 3 shows testing order data for the FE and SE. The results indicate minimal variances in the average central obstruction size, which suggests a high degree of consistency and similarity in the measurements obtained for both eyes. The large discrepancy observed at *N*_*f*_ = 7 is attributable to the floor effects associated with the lower spatial frequency of this specific condition, rather than a physiological difference. Specifically, the *N*_*f*_ = 7 stimulus results in a coarser entoptic pattern with reduced contrast cues, causing participant performance to approach the physical limit of the measurement (limit of measurable eccentricity). This creates a truncated range of data, where the thresholds are constrained by the testing parameters rather than reflecting true physiological variance. Consequently, the psychophysical thresholds for this condition exhibit higher variability and are more sensitive to minor fluctuations in attention or perception, explaining the apparent divergence between eyes that is not indicative of actual macular asymmetry. No formal statistical comparison was performed for the *N*_*f*_ = 7 condition. Due to the floor effects described above, the reliability of the threshold estimates for this specific condition is reduced, and statistical testing would not yield valid insights into true interocular differences.

**Table 2:** Central obstruction radius (i.e., *R*_*T*_ —retinal eccentricity of the threshold mask) for the various structured light-based entoptic images with visible azimuthal fringe numbers (*N*_*f*_) = 7, 12, 17 and 22 for the dominant (DE) and non-dominant (NDE) eyes.

Number of visible fringes (*n* of eyes)	Dominant eye	Non-dominant eye
	*R*_*T*_ mean ± SD [°]	
*N*_*f*_ = 7 (*n*_DE_ = 16, *n*_NDE_ = 19)	2.235 ± 0.82	3.284 ± 1.718
*N*_*f*_ = 12 (*n*_DE_ = 28, *n*_NDE_ = 27)	4.087 ± 1.806	4.299 ± 1.929
*N*_*f*_ = 17 (*n*_DE_ = *n*_NDE_ = 27)	5.836 ± 3.604	6.598 ± 3.134
*N*_*f*_ = 22 (*n*_DE_ = *n*_NDE_ = 28)	7.546 ± 4.604	7.072 ± 3.891

*SD* standard deviation.

**Table 3:** Central obstruction radius (i.e., *R*_*T*_ —retinal eccentricity of the threshold mask) for the various structured light-based entoptic images with visible azimuthal fringe numbers (*N*_*f*_) = 7, 12, 17 and 22 for the first (FE) and second (SE) eyes.

Number of visible fringes (*n* of eyes)	First eye	Second eye
	*R*_*T*_ mean ± SD [°]	
*N*_*f*_ = 7 (*n*_FE_ = 17, *n*_SE_ = 18)	2.762 ± 1.614	2.844 ± 1.35
*N*_*f*_ = 12 (*n*_FE_ = 27, *n*_SE_ = 28)	4.222 ± 1.676	4.161 ± 3.039
*N*_*f*_ = 17 (*n*_FE _= *n*_SE_ = 27)	6.194 ± 3.503	6.239 ± 3.293
*N*_*f*_ = 22 (*n*_FE_ = *n*_SE_ = 28)	7.648 ± 4.667	6.971 ± 3.799

*SD* standard deviation.

### Interocular Characteristics of the coMPOD Model

The study involved adjusting the spatial density and temporal frequency (*N*_*f*_ = 7, *ω* = 1.9 Hz; *N*_*f*_ = 12, *ω* = 3.8 Hz; *N*_*f*_ = 17, *ω* = 5.8 Hz; *N*_*f*_ = 22, *ω* = 7.7 Hz) with the central obstruction radius *R*_*T*_ (from Tables 2 and 3) of the SL entoptic pattern to calculate the coMPOD profile. The mean ± SD parameters for individual participant fits of the *α*-value for eye dominance revealed an average of 0.11 ± 0.06° for the DE and 0.11° ± 0.05 for the NDE. Additionally, the data for testing order effects showed that for the FE and SE, the values were 0.11 ± 0.05° and 0.10 ± 0.05°, respectively. As coMPOD measurement via SL is a novel technique, established normative ranges for the *α*-value in the general population are still being defined. The values observed in this healthy cohort provide a preliminary reference point for future studies. In both interocular variables, coMPOD *α*-values suggest no significant learning effect for the SE and no effect of eye dominance. A paired *t*-test comparing the *α*-values for the FE and the SE showed no significant difference (*t*(25) = 0.72, *p* = 0.48), indicating that a learning effect was not observed under the current testing conditions.

Considering the role of the *α*-value in the characterisation of coMPOD profile *M*(*r*) in Eq. 2, this variable was used to determine the correlation of interocular difference in the perception of SL-based stimuli, both for eye dominance and testing order effects.

**Fig. 2:**
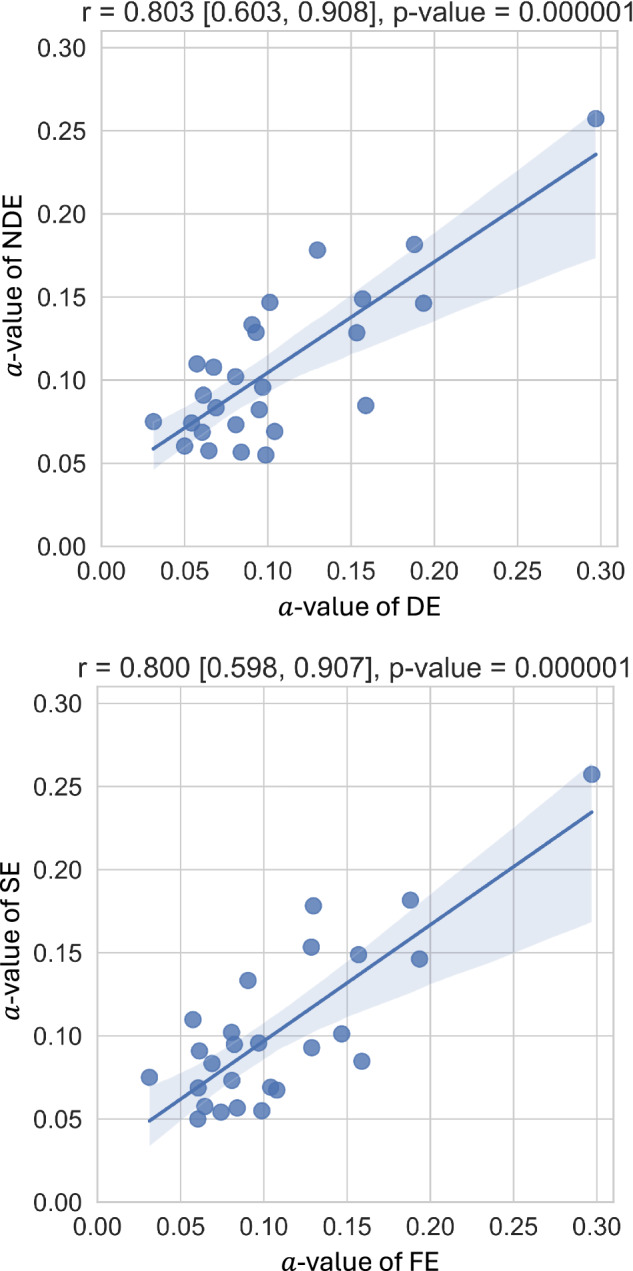
Correlation coefficient (*r*) and confidence interval (CI) for the beta value of the regression line and *p* value for the *α*-values of dominant (DE) vs non-dominant (NDE) eyes (top) and first (FE) vs second (SE) eyes (bottom).

**Fig. 3:**
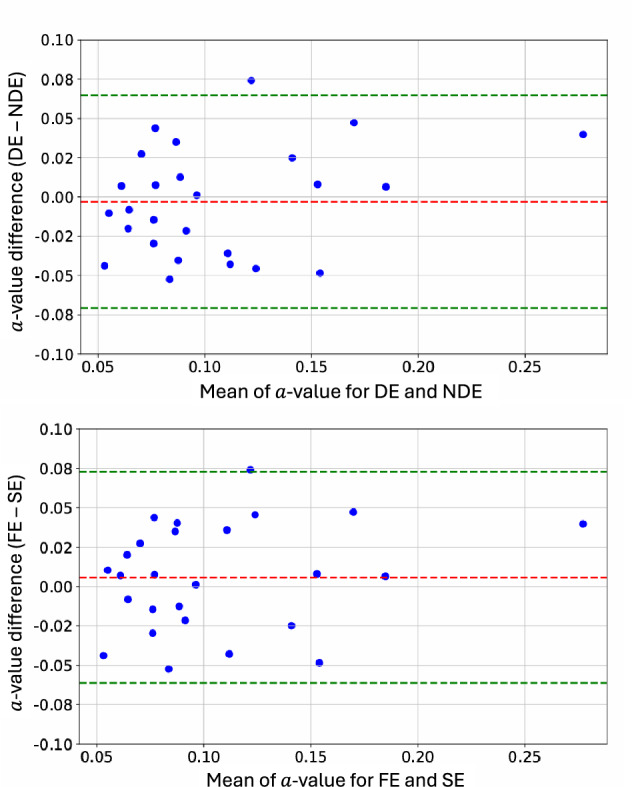
Agreement analysis for the circularly-oriented macular pigment optical density (coMPOD) *α*-value, where the *α*-value characterises the radial scale of retinal polarisation efficiency, determining the extent of measurable contrast from the fovea. Bland–Altman plot for dominant (DE) vs non-dominant (NDE) eyes (top) and first (FE) vs second (SE) eyes (bottom): interocular difference measurements against their mean, with the red dashed line representing the mean difference. The green dashed lines indicate the upper and lower limits of agreement, calculated as 1.96 times the standard deviation of the differences.

A total of 26 participants (52 eyes) were included in the correlation and Bland–Altman methods; two participants were excluded from the analysis due to the inability to model their binocular results accurately, as they had fewer retinal eccentricity thresholds for either of their eyes. Pearson correlation coefficient (*r*) for both interocular factors (eye dominance and testing order effects) was 0.80 (*p* < 0.01; Fig. 2). The Bland–Altman plot (Fig. 3) depicts the agreement between the measurements of *α*-value in the DE vs NDE and FE vs SE. The red dashed line represents the mean difference between the eyes, which is close to zero, indicating a minimal bias in the measurements. The upper and lower limits of agreement (green dashed lines) are approximately equal to 1.96 × SD (0.06). The mean difference for DE vs NDE was −0.01°, with 95% limits of agreement ranging from −0.07° to 0.06°. For FE vs SE, the mean difference was 0.01° with 95% limits of agreement from −0.06° to 0.08°. The majority of data points (95%) fall within the limits of agreement, suggesting good concordance of the interocular *α*-value and indication of the test-retest repeatability of this measurement.

## Discussion

The perception of SL stimuli in both of an individual’s eyes with similar interocular health indicators (visual acuity, contrast sensitivity, macular volume of HFL, MPOD and ocular biometry parameters) is invariant to ocular dominance [32, 33] and eye testing order. In addition, the eye dominance and testing order correlation plots displayed a strong positive correlation between the interocular *α*-value. Based on these results, it is appropriate to compare the measured coMPOD from each eye of a patient with a unilateral or asymmetric retinal condition [44–48]. These parameters of SL-based stimulus perception present a promising prospect for the future utilisation of this phenomenon in the assessment of the coMPOD profile in patients with various macular pathologies.

Although sufficient for initial observations, the sample size used here may not capture the full range of interindividual variability in SL-based stimulus perception. Future studies with larger and more clinically diverse populations are necessary to validate these findings. In addition, investigating polarisation perception in patients with macular disorders could provide further insights into the diagnostic potential of SL-based techniques. Another area for future research involves exploring the underlying mechanisms driving the observed consistency in polarisation perception. Understanding how the retinal polariser influences SL-based stimulus perception could enhance the development of more precise and targeted diagnostic tools.

The main clinical advantage of measuring the coMPOD profile compared with traditional assessments of central or peripheral MPOD is its ability to characterise the structural organisation and radial efficiency of Henle’s fibres. While traditional MPOD quantifies pigment density at specific loci, the coMPOD *α*-value provides a spatial profile that may be more sensitive to early structural disorganisation (e.g., in MacTel type 2) before significant pigment loss occurs. Nevertheless, it is important to qualify the interpretation of the coMPOD profile in the context of known inter-subject variability in MP spatial distributions [49]. Previous research has identified diverse MPOD profiles among healthy individuals, including decreasing exponential functions, distributions with a prominent shoulder at ~1° eccentricity and shallow trimodal distributions [49, 50]. Furthermore, specific subpopulations, such as those with a family history of AMD, may exhibit a foveal central ‘dip’ in MPOD despite normal peripheral levels [51, 52]. Since the coMPOD *α*-value is derived from the radial extent of perceivable entoptic fringes, it primarily reflects the spatial efficiency of the radial polariser effect (structural organisation of Henle fibres) rather than the absolute central density or total volume of the pigment. Consequently, while this technique offers a novel functional biomarker of macular structural organisation, sole reliance on radial extent may have limitations in detecting specific anomalies in central pigment density. Therefore, the coMPOD measurement should be viewed as a complementary tool to standard MPOD assessments, providing specific information regarding the radial integrity of the macula. The coMPOD-based method may be particularly advantageous for detecting early asymmetric pathologies where structural disorganisation precedes functional density loss. Current literature on normative ranges for coMPOD is evolving, but the consistency observed in this healthy cohort supports its potential as a reliable biomarker. Future studies are needed to establish definitive sensitivity and specificity values relative to conventional MPOD in diseased populations.

The promising prospects for deploying the entoptic phenomenon further as an early detection tool of macular diseases such as AMD, MacTel type 2 and pathologic myopia stem from its ability to provide objective, quantitative and selective measurements of MPs. By utilising this phenomenon, clinicians and researchers can potentially identify subtle changes in the macula associated with macular degeneration at a much earlier stage than traditional diagnostic methods allow. This could lead to improved patient outcomes by enabling timely interventions and treatments [53].

The study demonstrates that eye dominance and eye testing order do not influence the perception of SL-induced entoptic images significantly. The robustness of SL-based stimulus perception highlights the promising prospects for further deployment of SL in early detection tools for macular pathologies.

## Data Availability

The data that support the findings of this study are available on request from the corresponding author. The data are not publicly available due to privacy or ethical restrictions.
